# Modulation of Placental Breast Cancer Resistance Protein by HDAC1 in Mice: Implications for Optimization of Pharmacotherapy During Pregnancy

**DOI:** 10.1007/s43032-021-00773-2

**Published:** 2021-10-19

**Authors:** Chuan Wang, Dan Ma, Yimin Hua, Hongyu Duan

**Affiliations:** 1 Department of Pediatric Cardiology, West China Second University Hospital, Sichuan University, No. 20, Section 3, RenminNanLu Road, Chengdu, 610041 Sichuan China; 2 Cardiac Development and Early Intervention Unit, West China Institute of Women and Children’s Health, West China Second University Hospital, Sichuan University, Chengdu, Sichuan China; 3 Department of Pediatric Rehabilitation, West China Second University Hospital, Sichuan University, Chengdu, Sichuan China; 4 Key Laboratory of Birth Defects and Related Diseases of Women and Children (Sichuan University), Ministry of Education, Chengdu, Sichuan China; 5 Key Laboratory of Development and Diseases of Women and Children of Sichuan Province, West China Second University Hospital, Sichuan University, Chengdu, Sichuan China

**Keywords:** Placenta, Breast cancer resistance protein, HDAC1, Pharmacotherapy, Gestation, Epigenetic regulation

## Abstract

Breast cancer resistance protein (BCRP/*ABCG2*) is a critical drug efflux transporters by limiting drugs’ transplacental transfer rates. More investigations on the regulation of placental BCRP offer great promise for enabling pronounced progress in individualized and safe pharmacotherapy during pregnancy. Histone deacetylases (HDACs) play an important role in epigenetic regulation of placental genes. It was reported recently by us that HDAC1 was involved in placental BCRP regulation in vitro. The aim of this study was to further explore the effect of HDAC1 on placental BCRP expression and functionality in animals. Randomly assigned C57BL pregnant dams received intraperitoneal injections of a negative control siRNA or Hdac1 siRNA from embryonic day 7.5 (E7.5) to E15.5, respectively. At E16.5, glyburide (GLB), a probe for evaluating placental BCRP efflux functionality, was injected via the tail vein. Animals were sacrificed through cervical dislocation at various times (5–180 min) after drug administration. The maternal blood, placentas, and fetal-units were collected. GLB concentrations were determined by a validated high-performance liquid chromatography/mass spectrometry (HPLC-MS) assay. Real-time quantitative PCR (qRT-PCR), Western blot, and immunohistochemical (IHC) analysis were employed to identify mRNA/protein levels and localization of gene expressions, respectively. It was noted that Hdac1 inhibition significantly decreased placental Bcrp expression, with markedly increases of GLB concentrations and area under the concentration-time curve (AUC) in fetal-units. Particularly, the ratios of fetal-unit/maternal plasma GLB concentrations were also significantly elevated following Hdac1 repression. Taken together, these findings suggested that HDAC1 was involved in positive regulation of placental BCRP expression and functionality in vivo.

**Supplementary Information:**

The online version contains supplementary material available at 10.1007/s43032-021-00773-2.

## Introduction

Pharmacotherapy during pregnancy is increasingly common and often inevitable for treatment of various maternal and fetal conditions. Recent epidemiological studies have revealed that approximately 80% of pregnant women fill over-the-counter and/or prescription medications excluding vitamins and minerals [1, 2]. Depending on the intended therapeutic targets for medication (the mother, the fetus, or both), drug transfer across the placenta may be termed as either desired or undesirable. It is always difficult to balance a drug’s efficacy with its side effects when deciding on the treatment regimen in pregnant women. Therefore, detailed knowledge on transplacental passage of drugs and its influence factors is essential for efficient and safe therapy during pregnancy.

Accumulating evidence has suggested that ATP-binding cassette transporters (ABC transporters) in the placenta play a critical role in controlling drugs’ transplacental transfer rates [3]. Among them, breast cancer resistance protein (BCRP), encoded by *ABCG2* gene, is one of the most widely studied transporters in the placenta. BCRP is enriched on the maternal-facing surface of the placental syncytiotrophoblast where it actively effluxes a wide spectrum of clinically relevant drugs (e.g., anticancer, anti-human immunodeficiency virus drugs, hypoglycemics, antibiotics, anti-viral drugs) back to the maternal circulation [4]. A plethora of studies, to date, have illustrated that BCRP is a key player in controlling drugs’ transplacental transfer rates [4–6]. More investigations on the regulation of placental BCRP offer great promise for optimizing pharmacotherapy during pregnancy.

Recent studies have highlighted the potential importance of epigenetic effects on cellular proliferation, trophoblast differentiation/function, and adaptive responses to stress factors within the placenta [7]. Obviously, epigenetic regulatory mechanisms play essential roles in broad spectra of placental genes at the vulnerable time during embryonic development both in physiological and pathological conditions. Nonetheless, there has been little research assessing the roles of epigenetics in regulating placental drug transporters. As an important group of epigenetic-modifying enzymes, histone deacetylases (HDACs) could remove acetyl groups from the lysine tails of target proteins, altering chromatin conformation or activities of transcriptional factors, leading to a change in gene expression [8]. It has been reported recently by us that HDAC1 was involved in placental BCRP regulation in vitro [9]. However, whether HDAC1 was still engaged in this process in vivo needs to be further verified. Therefore, the aim of this study was to explore the effect of HDAC1 silencing on placental BCRP expression and functionality in animals. The data obtained in this study will expand the limited knowledge with regard to epigenetic regulation of placental BCRP and shed some light on controlling drug delivery across the placenta, which is imperative for optimization of therapeutic strategies during pregnancy.

## Materials and Methods

Randomly assigned C57BL pregnant dams received intraperitoneal injections of 0.3 mL saline containing a negative control siRNA (10 nmol each) or Hdac1-specific siRNA (10 nmol each) every 48 h from E7.5 to E15.5. The modified Hdac1 siRNA (2OMe+5Chol) sequences used for mice injection were as follows: Sense: 5′-GUUCUAUUCGCCCAGAUAA dTdT-3′; Anti-sense: 3′-dTdT CAAGAUAAGCGGGUCUAUU-5′. Prior to the sample collection at E16.5, glyburide (GLB) was injected via the tail vein at a dose of 100 μg/kg. At various times (5, 10, 20, 30, 40, 60, 120, and 180 min) after drug administration, animals (*n*=4–6 at each time point) were sacrificed under anesthesia via cervical dislocation. Maternal blood was collected via cardiac puncture. Placentas and fetal-units (comprised of fetus, all fetal membranes, and amniotic fluid) were quickly gathered. Real-time quantitative PCR (qRT-PCR), Western blot, and immunohistochemical (IHC) analysis were employed to identify mRNA/protein levels and localization of gene expressions, respectively. GLB concentrations in the maternal plasma, placenta, and “fetal-unit” were determined by a validated high-performance liquid chromatography/mass spectrometry (HPLC-MS) assay. GLB concentration of “fetal-unit” (ng/g) was presented below: value derived for “fetal-unit” homogenate (ng/mL) * the total homogenate volume (mL)/“fetal-unit” weight (g). For per dam, the average of individual fetus-unit concentration by litters was utilized for analysis. In a similar manner, GLB concentration of the placenta for per dam was assessed. GLB transplacental transfer was calculated as a ratio of “fetal-unit” concentration (ng/g) relative to maternal plasma concentration (ng/mL). The Bailer’s approach was employed to estimate the mean and standard error of mean (SEM) for area under the concentration-time curves (AUCs) of GLB in the maternal plasma and fetal-unit. Data were presented as means±SEM and analyzed by SPSS 17.0 version (SPSS, Chicago IL, USA). The significance of the difference between two groups was determined by the independent sample *t*-test. Multiply comparisons were made with analysis of variance (ANOVA) followed by Student’s *t*-test with the Bonferroni correction. A 2-tailed *P* value<0.05 was taken to be statistically significant. The Materials and Methods are shown in the “supplemental materials” in detail.

## Results

**Fig. 1:**
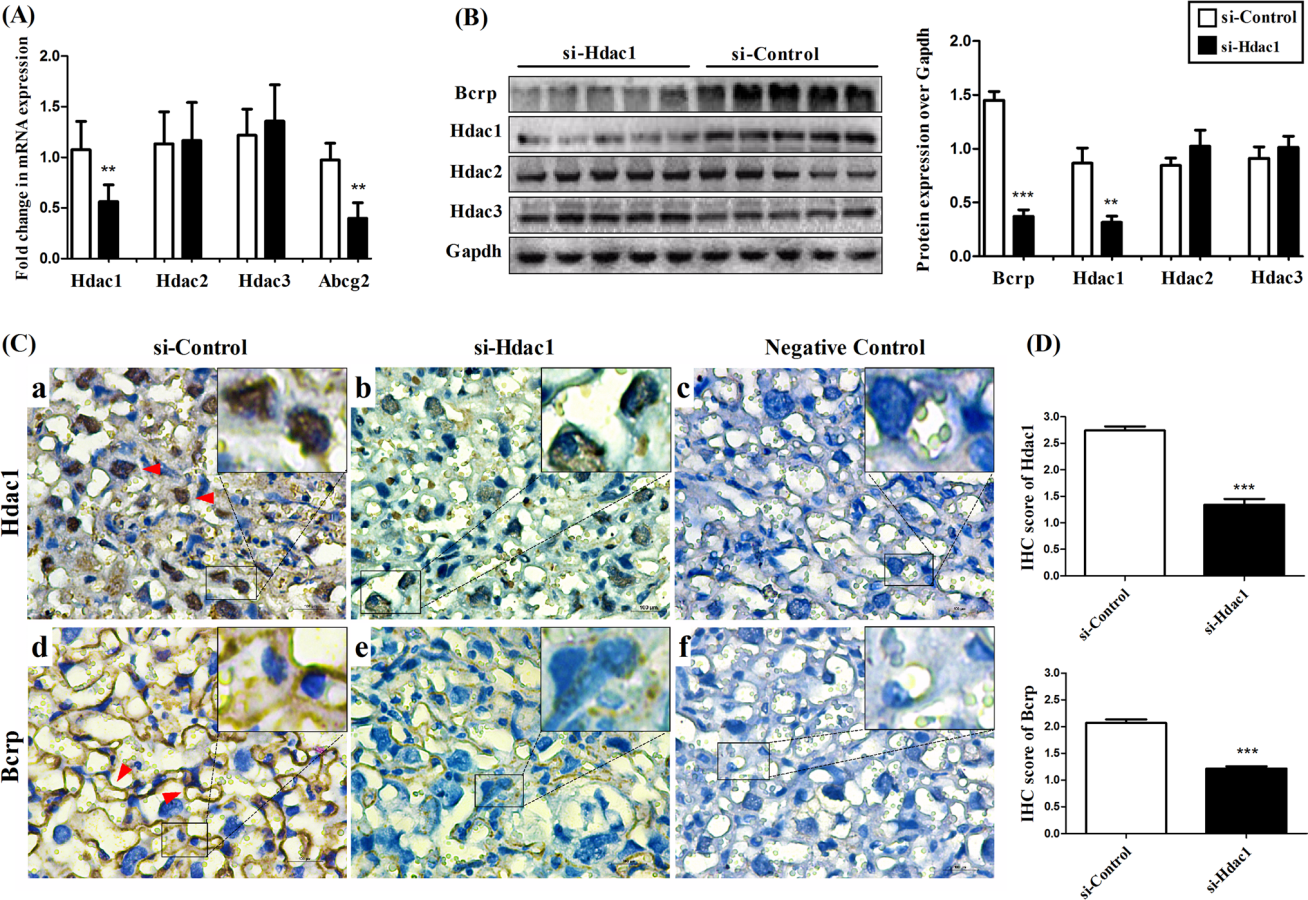
Impact of Hdac1 silencing on placental Bcrp expression in mice. Hdac1 siRNA or control siRNA was injected intraperitoneally every 48 h from E7.5 to E15.5. Mice were sacrificed at E16.5 and the placentas were collected. Hdac1/Hdac2/Hdac3/Bcrp mRNA (A) and protein levels (B/C) were determined by qRT-PCR, Western blot, and immunohistochemistry, respectively. The samples analyzed by Western blot were driven from the same experiment and that gels/blots were processed in parallel (B). Bcrp and Hdac1 protein staining were indicated in brown and by arrows. Negative staining control using mouse non-specific serum instead of primary antibody (C). Quantitative analysis using the IHC score was achieved by calculating the positively stained intensity and percentage of positive cells in immunohistochemistry images (3 random fields) (D). Two-tailed Student’s *t*-test was performed for data analysis. *n*=8 for each group. Scale bar=100 μm. Data were expressed as means±SEM. **P<0.01, ***P<0.001

As revealed in Fig. 1, Hdac1 siRNA intraperitoneal injection dramatically diminished Hdac1 mRNA (Fig. 1A) and protein expression (Fig. 1B) in the placenta as compared with the control group of same gestational age in mice *(P*<0.01). By contrast, Hdac2/3 mRNA and protein were not significantly affected in comparison with the control (*P*>0.05) (Fig. 1A/B). It was particularly noteworthy that repression of Hdac1 significantly depressed Bcrp mRNA and protein production (*P*<0.01) (Fig. 1A/B). Using IHC staining, Bcrp protein (in brown) was found to be mainly restricted to luminal membranes of syncytiotrophoblast, while Hdac1 protein (in brown) was predominantly confined to the nuclei. A consistent decline in staining intensity of Bcrp was detected following Hdac1 siRNA injection, but without impact on its tissue distribution (Fig. 1C). Next, IHC scores for Hdac1 and Bcrp were determined quantitatively using assessment of the percentage of stained cells combined with their staining intensities in immunohistochemistry images. The results demonstrated that there were dramatic decreases in the IHC scores of both Hdac1 and Bcrp in Hdac1 siRNA-treated mice as compared to those treated with the control (Hdac1: 1.34±0.11 vs. 2.74±0.08, *P*<0.001; Bcrp: 1.22±0.04 vs. 2.07±0.07, *P*<0.001) (Fig. 1D).

**Fig. 2:**
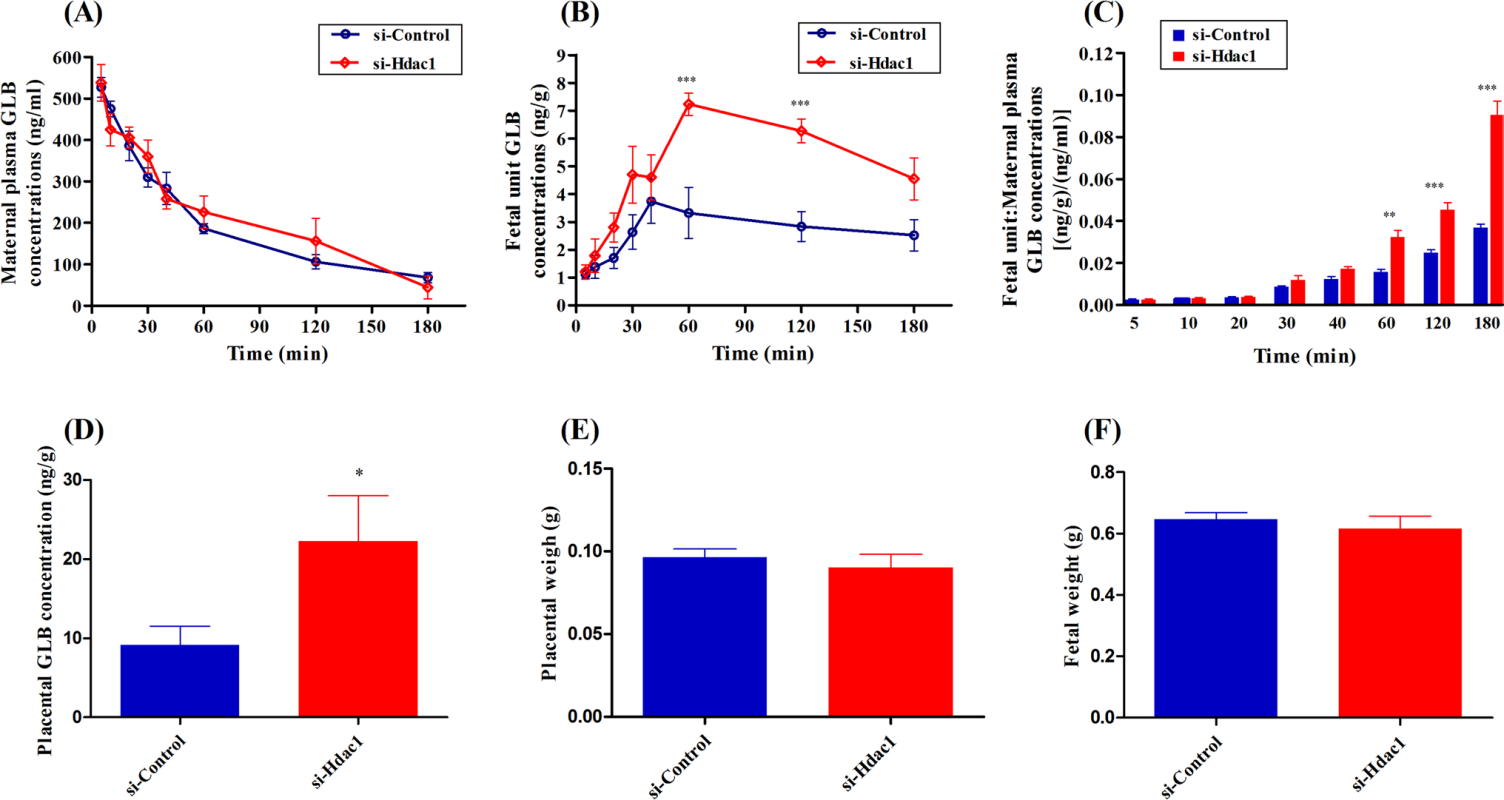
Impact of Hdac1 siRNA on placental Bcrp functionality (**A**–**C**), placental GLB disposition (**D**), placental weight (**E**), and fetal weight (**F**) in mice. Hdac1 siRNA or control siRNA was injected intraperitoneally every 48 h from E7.5 to E15.5. At E16.5, mice were sacrificed at various times (5–180 min) after GLB injection via the tail vein. The significance of the difference between two groups was determined by the independent sample *t*-test. Multiply comparisons were made with analysis of variance (ANOVA) followed by Student’s *t*-test with the Bonferroni correction. *n*=4–6/time point, *n*=8 for either control- or Hdac1-siRNA group. Data were expressed as means±SEM. *P<0.05, **P<0.01, ***P<0.001. GLB, glyburide

To further assess the regulation of Hdac1 on placental Bcrp function in vivo, GLB transplacental ratio was evaluated in pregnant mice. After administration of GLB, there were no significant differences in maternal plasma GLB concentrations at any given time points between the control- and Hdac1-siRNA groups (*P*>0.05) (Fig. 2A). Similarly, the maternal plasma AUCs_5–180 min_ of GLB between the two groups were comparable (*P*>0.05) (Table 1). The fetal-unit GLB concentrations in the control- and Hdac1-siRNA groups were low at early time points, and reached the maximum at approximately 40 and 60 min, respectively, with an overall significant difference (*P*=0.0303) (Fig. 2B). When making comparisons within the various time points, at 60 and 120 min, the mean fetal-unit GLB concentrations were dramatically elevated in the Hdac1 siRNA-transfected mice (greater than 2 times) compared with those in the controls (*P*<0.001). Consequently, the fetal-unit AUC_5–180 min_ of GLB in the Hdac1-siRNA group was significantly increased approximately 2-fold compared with the control (962.3 versus 490.8ng·min/g, respectively; *P*<0.001) (Table 1). It was noted that the fetal-unit/maternal plasma GLB concentration ratios in Hdac1 siRNA-transfected mice were generally greater than those in the controls, robust increases being observed at 60, 120, and 180 min (*P*<0.01) (Fig. 2C). Additionally, the fetal-unit/maternal plasma AUC ratio of GLB in the Hdac1 siRNA-transfected mice was approximately 1.75 times greater than that in the control (Table 1). No significant differences were found in placental weights (Fig. 2E) and fetal weights (Fig. 2F) between the control- and Hdac1-siRNA groups (*P*>0.05). However, significant increase of GLB accumulation was seen in placental tissues of Hdac1 siRNA-transfected mice (*P*<0.05) (Fig. 2D).

**Table 1:** Maternal plasma and fetal-unit AUCs (5–180 min) of GLB in the control- and Hdac1-siRNA pregnant mice after intravenous administration of the drug (100 μg/kg)

Parameter	Control siRNA	Hdac1 siRNA	*Z*_0_	*P*
Maternal plasma AUC_5–180min_ (ng min/mL)	31,943±2237	35,801±2646	1.11	>0.05
Fetal-unit AUC_5–180min_ (ng min/g)	490.8±55.3	962.3±59.8	5.79	<0.001
Fetal-unit/maternal plasma AUC ratio (%)	1.54	2.69		

The maternal and fetal-unit AUCs of GLB was estimated using the Bailer’s approach as described in the “Materials and Methods” section. The fetal-unit/maternal plasma AUC ratios were also presented. Data were expressed as means±SEM. *Z*_0_ and *P* values were calculated to assess the significance of the differences in the parameters between the control- or Hdac1-siRNA mice groups

To rule out compensatory alterations in placental gene expressions that could affect the disposition of GLB in vivo, the mRNA expressions of some best-described placental ABC transporters were compared between control- and Hdac1-siRNA placentas (Table 2). The majority of transcripts assessed indicated no significant alterations between two groups. The only exception here was *Abcc2*, showing a 1.8-fold increase in Hdac1- siRNA placentas (*P*<0.01).

**Table 2:** Gene expressions of best-described placental ABC transporters in Hdac1-siRNA-transfected mouse placentas

Name of genes	Fold change	*P*<0.05
Abcb1a (P-gp)	1.12	N
Abcb1b (P-gp)	1.01	N
Abcc1 (Mrp1)	0.82	N
Abcc2 (Mrp2)	1.80	Y
Abcc3 (Mrp3)	1.13	N
Abcc4 (Mrp4)	1.08	N
Abcc5 (Mrp5)	0.94	N

Data represented the mean fold-changes in Hdac1-siRNA-transfected placentas compared with control placentas. *n*=8 for each group. Two-tailed Student’s *t*-test was performed for data analysis. Y represented statistically significant differences between two groups (*P*<0.05)

## Discussion

Considering the limited understanding of epigenetic mechanisms in regulating placental BCRP, some further exploration with focus on classIHDACs was made on the basis of our previous study [9], which could provide some clinically references for the individualized and safe pharmacotherapy during pregnancy. It was demonstrated that inhibition of HDAC1 by HDAC1 siRNA intraperitoneal injection was capable of prohibiting BCRP expression and efflux functionality in vivo, without alteration of its tissue distribution. There was no impact on placental weights, fetal weights, and maternal plasma concentrations/AUC of GLB following HDAC1 repression. Once again, these findings strongly implied that HDAC1 was engaged in the positive regulation of placental BCRP expression and functionality.

Accumulating studies have demonstrated that numerous dietary bioactive compounds (e.g., sulforaphane, butyrate, epigallocatechin), which could be administered during pregnancy, are capable of inhibiting HDAC1 expression and activity [10]. Strikingly, these natural compounds hold great promise in terms of modulating gene expression upon alteration of protein acetylation status in the placenta [11, 12]. Since the target of pharmacotherapy during pregnancy is not only the mother but also the fetus or both, given the findings in this pilot study, at least to some extent, relevant clinical attention might be devoted to those dietary bioactive compounds, particularly when BCRP substrates are concomitantly administered during pregnancy. For instance, it is likely to maximize fetal drug exposure in a relatively safer manner when BCRP substrates and above dietary bioactive compounds are co-administrated during pregnancy (e.g., antiretroviral therapy to HIV-infected pregnant women for preventing vertical transmission of HIV from mother to fetus) [13], because transplacental transfer rates of BCRP substrates might be elevated and thereby promote the drug availability to the fetus. On the other hand, maternal diseases might be theoretically treated concomitantly by administering relatively lower quantities of BCRP substrates in such conditions, thus minimizing their adverse effects on the mother. Moreover, BCRP serves as removing its substrates already present in the fetal circulation back to the maternal space as well [6]. Therefore, downregulation of placental BCRP expression through HDAC1 could be profitable to further enhance the drug efficiency specific for the fetus in the context of decreasing drug clearance from the fetal-to-maternal direction. However, on account of the biological importance of HDAC1 in the placenta, maternal or fetal organs [8, 14–18], it is currently inappropriate to suggest HDAC1 inhibitors can be used therapeutically in pregnancy when BCRP decreases are desired. More studies should be further carried out to verify the safety of HDAC1 inhibition, particularly in the context of the fetal development and maternal health. Meanwhile, the accurate molecular network of HDAC1 governing placental BCRP surely warrants further clarification, which might identify more downstream regulatory targets and provide some safer regulatory approaches of placental BCRP during pregnancy.

The maternal plasma concentrations and AUCs_5–180 min_ values of GLB in the control- and Hdac1-siRNA-transfected mice were comparable (Table 1), suggesting that Hdac1-mediated placental Bcrp inhibition had only a negligible impact on the systemic clearance of GLB in the pregnant mouse. Based on high expression of Bcrp in the maternal organs of mice responsible for drug clearance (e.g., small intestine, kidney, and liver) [19], Hdac1 might regulate Bcrp expression or function in a tissue-specific manners. When comparing the fetal-unit concentrations of GLB, a delay in achieving peak concentrations in Hdac1 siRNA-transfected was noted, indicating that repression of placental Bcrp did not accelerate penetration of GLB. However, we still observed increased absolute and maternal plasma normalized concentrations of GLB in the “fetal-unit” following Hdac1 siRNA injection. Therefore, Hdac1-mediated alterations in the expression of placental Bcrp highly probably affected the fetal exposure of GLB.

Because of co-localization of placental ABC transporters and their overlap in substrate profiles, they may compensate for the downregulated/inhibited one, conferring absence of a clinically important change in the transplacental transport of a drug substrate [3]. Hence, it is also important to observe any compensatory transcriptional changes in other transporters following the downregulation of Bcrp. It was noted that Hdac1-siRNA-transfected placentas manifested a similar transcriptional profile of the best-described ABC transporters to those of controls, including those transporters implicated as minor players in GLB transportation, namely P-gp (*Abcb1a* and *Abcb1*), Mrp1 (*Abcc1*), and Mrp3 (*Abcc3*) [20]. Of note, *Abcc2*, the only exception here, showed a significantly higher mRNA level in placental tissues obtained from Hdac1-siRNA-transfected mice. Since an upregulation of *Abcc2* should contribute to decrease, rather than increase, the placental and fetal accumulation of GLB, this transporter did not seem to be a major player in placental transport of GLB, which was consistent with prior reports [3–5]. However, the ultimate impact on fetal drug accumulation could be varied with other xenobiotics, determined by their relative affinity to each transporter. Further work is needed to fully characterize the overall impact of placental BCRP following HDAC1 inhibition on drugs’ transplacental transfer rates.

Some limitations of the present study must be addressed. It was reported that placental BCRP expression decreased from midstage to the end of gestation. Because such variability could contribute to the gestational age-dependent alterations in fetal exposure to GLB [20], the extrapolation of these data to the entire gestational stage is possibly difficult. Moreover, placental BCRP also facilitates transportation of physiological substrates apart from drugs (e.g., sulfate conjugates and folic acid) [4], and further investigation will, therefore, be required to clarify alterations in the biodistribution of these compounds and its ultimate impact on fetal/placental development following placental BCRP inhibition. Additionally, it was observed that placental disposition of GLB was significantly elevated in the Hdac1 siRNA-transfected mice. Changes in Bcrp alone cannot fully explain these findings. Whether the observed phenomenon is resulted from a combination of the presumed effects of Bcrp or/and other transporters remains unknown. Despite these limitations, we made some preliminary exploration of placental BCRP regulation in the context of expression and function from the perspective of epigenetics, which was currently largely unexplored in the research field. These data obtained from current pilot experiments might expand the limited information regarding epigenetic regulation of placental BCRP and provide some references for individualized and safe pharmacotherapy during pregnancy.

## Supplementary Information

ESM 1 (DOC 64 kb)

ESM 2 (DOCX 16 kb)

## Data Availability

The datasets used and/or analyzed during the current study are available from the corresponding author on reasonable request.

## Abbreviations

BCRP: breast cancer resistance protein
HDAC: Histone deacetylase
GLB: glyburide
HPLC-MS: high-performance liquid chromatography/mass spectrometry
qRT-PCR: real-time quantitative PCR
IHC: immunohistochemical
AUC: area under the concentration-time curve (AUC)
ABC transporters: ATP-binding cassette transporters
SEM: means±standard error of mean
Mrp: Multidrug resistance-associated protein
