# Supplementary material for: Modulation of Placental Breast Cancer Resistance Protein by HDAC1 in Mice: Implications for Optimization of Pharmacotherapy During Pregnancy
Source: Reprod Sci. 2021 Oct 19;28(12):3540–6. doi: 10.1007/s43032-021-00773-2 (PMC8580892; doi:10.1007/s43032-021-00773-2)
Supplement: Supplementary file 1 — (DOC 64 kb) [file 43032_2021_773_MOESM1_ESM.doc]

**Materials and methods**

**Animals**

The healthy and adult SPF class C57BL mice aging 8~10 weeks (weigh 21 to 26 g) were purchased from Sichuan University Animal Institution. Animals were housed in groups of five, had free access to food (a standard diet) and water, and maintained on a 12 h light/dark cycle (light phase: 08:00~20:00) in a temperature-controlled environment (21±2℃). Female mice were mated with the male at 5:00 PM and were inspected for the vaginal plug at 08:00 AM in the next morning. Pregnancy was defined after the presence of a vaginal plug, and designated as embryonic day 0.5 (E0.5). All the procedures were performed in accordance with the National Institutes of Health Guide and with the approval of the Sichuan University Committee for the Care and Use of Laboratory Animals.

**HDAC1 siRNA intraperitoneal injection and sample collection**

Based on the results of siRNA transfection *in vitro* experiments, demonstrating only HDAC1 was involved in placental BCRP regulation [1], a specific siRNA for Hdac1 (siM190917022742, GuangZhou RiboBio. Co., China) and a negative control siRNA (siM121010150938, GuangZhou RiboBio. Co., China) were constructed for animal studies. On account of the indispensable role of Hdac1 in cell differentiation, proliferation and morphogenesis (Hdac1 deletion at early stage of embryogenesis might result in embryonic lethality and devastating effects on placental functions) [2-4], as well as the higher expression and functional activity of Bcrp at mid to the end gestational stage in rodent placentas [5], randomly assigned pregnant dams received intraperitoneal injections of 0.3 mL saline containing a negative control siRNA (10 nmol each) or Hdac1-specific siRNA (10 nmol each) every 48 h from E7.5 to E15.5. The dose and interval of siRNA administration were selected according to the reference data [6,7]. The modified Hdac1 siRNA (2OMe+5Chol) sequences used for mice injection were as follows: Sense: 5'-GUUCUAUUCGCCCAGAUAA dTdT-3'; Anti-sense: 3'-dTdT CAAGAUAAGCGGGUCUAUU-5'.

Glyburide (GLB) has been extensively used as a pharmacological probe for evaluating placental BCRP efflux functionality [8]. Thus, GLB was administered at a dose of 100 μg/kg via the tail vein before the sample collection at E16.5. Dams were sacrificed via cervical dislocation at various times (5, 10, 20, 30, 40, 60, 120, and 180 min) after drug administration. Maternal blood was collected via cardiac puncture. Plasma was separated and stored at -80 ℃ for further analysis. Placentas were quickly removed, weighed, and stored at -80 ℃ for further mRNA/protein expression and GLB concentration analysis. Fetal-units (comprised of fetus, all fetal membranes and amniotic fluid) were collected and weighed. To determine GLB concentrations in fetal-units and placentas, either four litters or placentas per dam were arbitrarily collected from both sides of the uterus, and averaged per dam to provide a mean of the concentration used for statistical analysis. From the remainder of the litters, fetuses and placentas were collected and weighted at the time of dissection, immediately applied to next step tests or stored at -80 ℃ until use. Subsequently, a volume of deionized water equivalent to the “fetal-unit” or placenta weight was added, “fetal-unit” or placenta being homogenized. Then, the final volumes of “fetal-unit” or placenta homogenates were gathered. The GLB concentrations in the maternal blood, placenta and “fetal-unit” were determined by a validated high-performance liquid chromatography/mass spectrometry (HPLC-MS) assay. For per dam, GLB concentration of the “fetal-unit” (ng/g) was presented below: value derived for “fetal-unit” homogenate (ng/mL) * the total homogenate volume (mL)/“fetal-unit” weight (g); the GLB concentration of the placenta (ng/g) was present in a similar manner. Finally, GLB transplacental transfer was calculated as a ratio of “fetal-unit” concentration (ng/g) relative to maternal plasma concentration (ng/mL).

**Real-time quantitative PCR analysis**

Total placental RNA was isolated and purified using the Trizol reagent (Invitrogen, Life technologies, Carlsbad, CA). RNA concentration and purity were assessed by a A260/A280 ratio spectrophotometrically using the Nanodrop_2000 instrument (Thermo Scientific). RNA integrity was determined by agarose gel electrophoresis using the 28S/18S rRNA ratio. RNA (1 μg) was reverse transcribed into cDNA using PrimeScript^TM^ RT Reagent Kit with gDNA eraser (RR0047A, Takara, Japan) according to the manufacturer's instructions.

Amplification of cDNA was performed with SsoFast EvaGreen Supermixture (Bio-Rad Laboratories, Hercules, CA) using 5 μL reaction mixture, 3 μL nuclease-free H_2_O, 0.5 μL forward primer, 0.5 μL reverse primer and 1 μL cDNA in a final reaction volume of 10 μL. The cycling conditions were as follows: initial denaturation at 95 ℃ for 3 min, followed by 39 cycles of 30 s at 95 ℃, 10 s at 58 ℃, and a continuous melt curve from 65-95 ℃. A validation experiment had been performed in which equivalent amounts of cDNA were used. The stability of *Gapdh* expression between the control group and the treatment group guaranteed its use as an appropriate endogenous control for normalization. Additionally, we have ascertained the efficiencies of amplifications for all genes in our study, which were consistent across a range of template concentrations. All the slope of the amplification efficiency curves were more than 95% and efficiencies for the target genes and the internal control (*Gapdh*) were approximately equal (0.965-0.978). All samples were amplified in triplicates. Gene expression was represented for the cycle threshold value (CT) by the mean of triple tests. Data were normalized to expression of *Gapdh* and calculated through 2^-△△Ct^ method. The primer sequences specific for target genes were available at Supplementary Table 1.

**Western blot analysis**

Samples were lysed in RIPA (P0013B, Beyotime, China) containing complete protease inhibitor cocktail (P8340, Sigma-Aldrich) for 20 min at 4 ℃ and centrifuged at 12,000 g for 5 min at 4 ℃. Supernants were analyzed for protein concentration by enhanced BCA protein assay kit (P0010S, Beyotime, China) following manufacturer’s protocol. Cell lysates were boiled in 4×sample buffer for 5 min and 50 μg protein/lane was subjected to 8% SDS-polyacrylamide gel, followed by blotting onto polyvinylidene difluoride membranes (Millipore, Bedford, MA). After blocking for 60 min with 5% nonfat milk in Tris base buffer containing 0.1% Tween 20 (TBST), membranes were incubated overnight at 4 ℃ with primary antibodies against Hdac1 (dilution 1:1000; 10197-1-AP, Proteintech)/Hdac2 (dilution 1:1000; 12922-3-AP, Proteintech)/Hdac3 (dilution 1:500; 10255-1-AP, Proteintech)/Bcrp (dilution 1:1000; ab130244, Abcam) and Gapdh (dilution 1:500; CW0100A, CWBIO). Following three times of washing with TBST, the membranes were reacted with horseradish peroxidase-conjugated goat anti-mouse/goat anti-rabbit immunoglobulin G (IgG) secondary antibodies (dilution 1:2500) for 2 h at room temperature. Washed thrice in TBST, the immunoreactive bands were visualized by enhanced chemiluminescene detection system. The protein band intensity was quantified by software Gelpro32 and normalized against the Gapdh as an internal control.

**Immunohistochemistry staining of placental HDAC1 and BCRP**

Fresh placental tissues were fixed in paraformaldehyde (4%) for at least 12 h, and then dehydrated progressively in 60%, 70%, 80% and 100% ethanol for 1 h, respectively. After xylene treatment, tissues were embedded in paraffin and sectioned at 5 μm. Subsequently, paraffin sections were dewaxed in xylene, and rehydrated in ethanol in descending gradients. Endogenous peroxidase activity was quenched with 3% hydrogen peroxide solution in absolute methanol for 20 min at room temperature, and then rinsed in PBS. Heat-induced antigen retrieval was performed by boiling the tissue sections in citrate buffer for 10 min, followed by several washing in PBS. The sections were blocked by a room temperature incubation with blocking serum for 2 h. Samples were incubated overnight at 4 ℃ with primary antibody specific for Hdac1 (10197-1-AP, Proteintech, 1:50 dilution) and Bcrp (ab207732, Abcam, 1:4000 dilution). Negative controls included incubation with PBS. Following several washing steps with PBS, the slides were incubated with goat anti-rabbit secondary antibody-HRP (ab6721, Abcam, 1:1000 dilution). The color reaction was developed by diaminobenzidine. Slides were counterstained with hematoxylin, dehydrated, and coverslipped. The slides were viewed and photographed using the microscope (Nikon, Tokyo, Japan).

**Immunohistochemical (IHC) scoring**

3 random-view fields of Hdac1 and Bcrp immunostained sections in each sample were obtained under 40X magnification. The positively stained intensity and percentage of positive cells in each image were quantified by Image J version 1.44 software. The detailed quantitative procedures used in this study were based on the protocol as previously described [9]. In brief, the original images were converted to 8-bit images before immunopositive staining was qualified through color deconvolution. Positive particles were detected on a black-and-white scale. Three montages of each image were produced for choice and determination of threshold and accurate measurements. ‘Create selection’ tool was used to mark the specific areas for automated analysis, and the positive signal area was measured by pixel-by-pixel analysis. Finally, the full profile with positively stained intensity and percentage of positive cells was displayed. Subsequently, the intensity score was assigned as 0 for negative, 1 for low positive, 2 for positive, and 3 for high positive. The following equation was used to assess the H-score of the target proteins in an individual image: H-score = (% of percentage of lowly-positive cells×1) + (% of percentage of positive cells×2) + (% of percentage of highly-positive cells×3). The average H-score of multiple images captured at different field of view of the same sample was considered to be the IHC score for each sample.

**HPLC-MS assay**

GLB concentrations in the maternal plasma, placenta, and “fetal unit” homogenates were performed using a validated HPLC-MS assay. In brief, to determine maternal plasma GLB concentrations, the samples were processed as described below. To every 100 μL maternal plasma, 20 μL working internal standard solution (glimepiride, 400 ng/mL) was added in a 13×100-mm borosilicate glass culture tube. Subsequently, 10 μL of 2 mol/L HCl and 1.5 mL n-hexane/methylene chloride (1:1, v/v) were added in each plasma sample for extraction of GLB and glimepiride. Samples were vortexed 1 min and centrifuged at 3000 rpm for 5 min. Supernatants were transferred to disposable clean glass tubes and evaporated to dryness under a nitrogen stream at 40 ℃. The dried residue for each sample was reconstituted in 100 μL initial mobile phase, and 20 μL of each sample was injected for HPLC-MS analysis. A calibration curve was prepared identically using human plasma as a matrix with a dynamic range of 0.5~1000 ng/mL for GLB.

To determine the GLB concentrations of placenta and “fetal unit” homogenates, the samples were processed as described below. To every 500 μL tissue homogenates, 20 μL working internal standard solution (glimepiride, 400 ng/ml) was added in a 13×100-mm borosilicate glass culture tube. Subsequently, 50 μL of 2 mol/L HCl, 4 mL n-hexane/methylene chloride (1:1, v/v) were added in each sample for extraction of GLB and glimepiride. The mixture was vortex-mixed for 1 min, and then centrifuged at 3000 rpm for 5 min. The upper organic layer was transferred to a disposable clean glass tube, and evaporated to dryness under a nitrogen stream at 40 ℃. The residue was reconstituted in 100 μL 1% formic acid in methanol, and 20 μL of each sample was injected for HPLC-MS analysis. A calibration curves was generated by adding GLB to the blank tissue homogenate (500 μL) to give final concentrations ranging from 0.5~500 ng/mL.

Separation of GLB and glimepiride was achieved using a Zorbax SB-C8 analytical column equipped with gradient elution. The mobile phase was consisted of water and methanol at pH 6.0, both containing 0.5 mM ammonium formate. The flow rate was set at 0.4 mL/min. At time 0, the mobile phase was 20% (v/v) methanol and 80% (v/v) water. The column was pre-equilibrated for 5.4 min, and the total run time was 15 min. The following parameters were set to optimize detection sensitivity: fragmentation voltage, 115 V; capillary voltage, 3500 V; drying gas temperature, 350 °C; nitrogen drying gas flow rate, 12 L/min; and nebulizer pressure, 25 psi. Blank human plasma spiked with GLB was used for quality control samples for maternal plasma, placenta, and “fetal unit” homogenate analyses.

**Data analysis of GLB disposition**

The Bailer’s approach was employed to estimate the mean and standard error of mean (SEM) for area under the concentration-time curves (AUCs) of GLB in the maternal plasma and fetal-unit [10, 11]. The following equations were used to asssess parameters of GLB disposition:

*ACU* indicated the mean of AUC. Where *m* indicated the number of time points in the time course experiment. *C_i_* and *t_i_* were concentration and time, respectively. *E(C_i_)* indicated the mean of the concentration at time *t_i_*, and defined as:

*V(AUC)* indicated the variance of mean AUC. where *n_i_* indicated the number of concentration data points at time *t_i_*. *V(C_i_)* indicated the variance of the concentration at time *t_i_* , and defined as:

The SEM of AUC was then given as the square root of *V(AUC)*.

The following equation was used to assess the statistically significant difference of AUCs between the control- and Hdac1-siRNA groups:

Where*φ_1_* and *φ_2_* inidicated the mean of a AUC, and *SEM_1_* and *SEM_2_* were the standard errors of a AUC, respectively in two animal groups. If *Z_0_*＞1.96 and *P*＜0.05, the difference of AUCs between the control- and Hdac1-siRNA groups was considered to be statistically significant.

**Statistical analysis**

Values for all data were expressed as means±SEM and were analyzed using SPSS 17.0 version (SPSS, Chicago IL, USA). The significance of the difference between two groups was assessed using the independent sample *t*-test. Multiply comparisons were made with analysis of variance (ANOVA) followed by Student’s *t*-test with the Bonferroni correction. Statistical significance was considered when a 2-tailed *P* value＜0.05.

**References**

1. Duan H, Zhou K, Zhang Y, Yue P, Wang T, Li Y, et al. HDAC1 was involved in placental breast cancer resistance protein regulation in vitro: A preliminary study. J Cell Mol Med. 2019; 23 (8): 5818-5821. doi: 10.1111/jcmm.14414

1. Montgomery RL, Davis CA, Potthoff MJ, Haberland M, Fielitz J, Qi X, et al.. Histone deacetylases 1 and 2 redundantly regulate cardiac morphogenesis, growth, and contractility. Genes Dev. 2007; 21 (14): 1790-1802. doi: 10.1101/gad.1563807
2. Maltepe E, Bakardjiev AI, Fisher SJ. The placenta: transcriptional, epigenetic, and physiological integration during development. J Clin Invest. 2010; 120 (4): 1016-1025. doi: 10.1172/JCI41211
3. Lagger G, O'Carroll D, Rembold M, Khier H, Tischler J, Weitzer G, et al. Essential function of histone deacetylase 1 in proliferation control and CDK inhibitor repression. EMBO J. 2002; 21 (11): 2672-2681. doi: 10.1093/emboj/21.11.2672
4. Mao Q. BCRP/ABCG2 in the placenta: expression, function and regulation. Pharm Res. 2008; 25 (6): 1244-1255. doi: 10.1007/s11095-008-9537-z
5. Dan C, Jinjun B, Zi-Chun H, Lin M, Wei C, Xu Z, et al. Modulation of TNF-α mRNA stability by human antigen R and miR181s in sepsis-induced immunoparalysis. EMBO Mol Med. 2015; 7 (2): 140-157. doi: 10.15252/emmm.201404797
6. Hou J, Zhou Y, Zheng Y, Fan J, Zhou W, Ng IO, et al. Hepatic RIG-I predicts survival and interferon-α therapeutic response in hepatocellular carcinoma. Cancer cell. 2014; 25 (1): 49-63. doi: 10.1016/j.ccr.2013.11.011
7. Staud F, Cerveny L, Ceckova M. Pharmacotherapy in pregnancy; effect of ABC and SLC transporters on drug transport across the placenta and fetal drug exposure. J Drug Target. 2012; 20 (9): 736-763. doi: 10.3109/1061186X.2012.716847
8. Jensen EC. Quantitative analysis of histological staining and fluorescence using ImageJ. Anat Rec (Hoboken). 2013; 296 (3): 378-381. doi: 10.1002/ar.22641
9. Bailer AJ. Testing for the equality of area under the curves when using destructive measurement techniques. Pharmacokinet Biopharm. 1988; 16 (3): 303-309. doi: 10.1007/BF01062139
10. Takemoto S, Yamaoka K, Nishikawa M, Takakura Y. Histogram analysis of pharmacokinetic parameters by bootstrap resampling from one-point sampling data in animal experiments. Drug Metab Pharmacokinet. 2006; 21 (6): 458-464. doi: 10.2133/dmpk.21.458
