# Supplementary material for: Modulation of Placental Breast Cancer Resistance Protein by HDAC1 in Mice: Implications for Optimization of Pharmacotherapy During Pregnancy
Source: Reprod Sci. 2021 Oct 19;28(12):3540–6. doi: 10.1007/s43032-021-00773-2 (PMC8580892; doi:10.1007/s43032-021-00773-2)
Supplement: Supplementary file 2 — (DOCX 16 kb) [file 43032_2021_773_MOESM2_ESM.docx]

**Supplementary Table 1** Primer sequences used for Real-time quantitative PCR

| Name of genes | Forward (5'-3') | Reverse (5'-3') |
| --- | --- | --- |
| Hdac1 | GAACTACCCACTGCGAGACG | ACAGGGAATCTGAGCCACAC |
| Hdac2 | GAAGGTGAAGGAGGTCGTAGG | AGGGTTGCTGAGTTGTTCTGA |
| Hdac3 | TGGTGGGAAGGA AAGTATGG | TGAGAGGGACAATCATCAGG |
| Abcg2 | GGCGGAGGCAAGTCTTCGTTG | TGGGCAGGTTGAGGTGCTCCAT |
| Abcb1a | GCTTACAGCCAGCATTCTCCGTAA | CCCTTTCACTTGAGCAGCATCGTT |
| Abcb1b | AAGGCAAGGGCTGTTAAAGG | TGTTTGTTTGTTTGTTTCCGATTT |
| Abcc1 | TGAGTGTGCAGAAGGTGGAG | ACCCGCGTGTAGTCCATTAT |
| Abcc2 | CTGAGTGCTTGGACCAGTGA | CAAAGTCTGGGGGAGTGTGT |
| Abcc3 | CGCTCTCAGCTCACCATCAT | GGTCATCCGTCTCCAAGTCA |
| Abcc4 | CCAGACCCTCGTTGAAAGAC | TGAAGCCGATTCTCCCTTC |
| Abcc5 | AGGGCAGCTTGTGCAGGTGG | TGCTGTTCCCGCTTCCTTGCT |
| Gapdh | CCCATCACCATCTTCCAGGAG | GTTGTCATGGATGACCTTGGC |
